# A Virtual Mental Health Clinic for University Students: A Qualitative Study of End-User Service Needs and Priorities

**DOI:** 10.2196/mental.3890

**Published:** 2015-02-11

**Authors:** Louise Farrer, Amelia Gulliver, Jade KY Chan, Kylie Bennett, Kathleen M Griffiths

**Affiliations:** ^1^ National Institute for Mental Health Research The Australian National University Acton Australia

**Keywords:** university, student, mental health, online, qualitative

## Abstract

**Background:**

Help seeking for mental health problems among university students is low, and Internet-based interventions such as virtual clinics have the potential to provide private, streamlined, and high quality care to this vulnerable group.

**Objective:**

The objective of this study was to conduct focus groups with university students to obtain input on potential functions and features of a university-specific virtual clinic for mental health.

**Methods:**

Participants were 19 undergraduate students from an Australian university between 19 and 24 years of age. Focus group discussion was structured by questions that addressed the following topics: (1) the utility and acceptability of a virtual mental health clinic for students, and (2) potential features of a virtual mental health clinic.

**Results:**

Participants viewed the concept of a virtual clinic for university students favorably, despite expressing concerns about privacy of personal information. Participants expressed a desire to connect with professionals through the virtual clinic, for the clinic to provide information tailored to issues faced by students, and for the clinic to enable peer-to-peer interaction.

**Conclusions:**

Overall, results of the study suggest the potential for virtual clinics to play a positive role in providing students with access to mental health support.

## Introduction

### Mental Health Problems and University Students

There is growing recognition of the high rates of mental health problems experienced by young adults attending universities. University students experience higher rates of psychological distress and mental disorders than their nonstudent peers [1-3]. Among US college students, approximately 50% will experience a mental disorder during a 12-month period [4].

However, help seeking by university students for mental health problems is very low. In a large US study, only 18% of students with a past-year mental disorder received treatment [4]. Identified barriers to help seeking for mental health problems in students are varied and include stigma [5,6]; lack of knowledge about services and their location [6,7]; failure to perceive themselves as in need of mental health support [8]; and lack of time [8]. Despite the provision of free short-term counseling at most university campuses, uptake of these services is far lower than would be expected on the basis of need. A US study found that only 12.7% of students with a mental health problem sought help from university campus services [9]. Moreover, many counseling services have resourcing constraints that limit the numbers of students that they can see, which may result in long waiting lists for the students who do seek assistance from these services [10].

### Web-Based Interventions

Evidence-based programs delivered via the Internet have the potential to broadly disseminate interventions to at-risk groups [11]. Several reviews and meta-analyses have demonstrated that Web-based interventions are effective in reducing symptoms of depression and anxiety in both adults [12,13] and university students [14]. Web-based interventions offer several advantages over face-to-face interventions, including 24-hour accessibility, anonymity, and a number are provided to end-users without the requirement for clinician support. These features may be particularly appealing to young people who face time pressures [15], fear stigma [16], and already have a high rate of engagement with the Internet [17].

Virtual clinics have been proposed as a model for providing streamlined, continuity of care for chronic physical and mental health conditions [18,19]. Virtual clinic models emphasise self-management, place the user at the center of their own care, and often incorporate several elements such as information, peer-to-peer support, facilitated access to professionals, and symptom screening and monitoring tools. In its role as a partner in the Australian Government funded Young and Well Cooperative Research Centre, the National Institute for Mental Health Research is currently developing a university virtual clinic, a comprehensive, Internet service targeting mental disorders in tertiary students. The present study was conducted as an initial phase of a larger user-centered development process to design, develop, and evaluate the university virtual clinic. Consulting end-users in the development of interventions allows appropriate tailoring of services to the target population and fosters a sense of empowerment and ownership that may improve uptake of and engagement with services [20]. Qualitative methods such as focus groups have been used in health services research to access the perspectives of consumers and end-users. Focus groups are particularly suited to assessing health needs and generating data to plan and develop effective interventions [21]. The aim of the present study was to conduct focus groups with university students to obtain input on potential functions and features of a university virtual clinic.

## Methods

### The Focus Groups

There were four focus groups that were conducted with students from the Australian National University (ANU) in November and December 2012. Each focus group consisted of four or five participants (n=5, 5, 4, 5) with a total of 19 participants. There were three groups that were conducted on the ANU campus, and one group was conducted at an off-campus university residential hall. Ethics approval was granted by the Australian National University Human Research Ethics Committee (2012/520). Cinema vouchers were offered to participants in recognition of their time and involvement in the focus groups.

### Participant Recruitment

Several methods were used to recruit participants. First, email invitations for a focus group on “student mental health and online programs” were sent to a list of students who had previously expressed interest in participating in a mental health research initiative and provided their contact details to the researchers at a mental health awareness event. Participants were also encouraged to invite other students to participate. Second, the researchers contacted the senior resident of a university residential hall for assistance in advertising the focus group to their students. The method of recruiting through the residential hall was most effective because the focus groups were directly and actively targeted to a captive group of students by someone who had a preexisting relationship with them. Recruitment of new participants was discontinued when consistencies emerged in the data. Following the fourth focus group, the facilitators met to review the notes taken during the groups by one of the facilitators, and to discuss the data obtained. The facilitators mutually agreed that data saturation had been achieved following the fourth focus group.

### Development of Focus Group Questions and Procedure

Focus group questions were developed by the researchers to investigate broad and specific issues relevant to the development of a virtual mental health clinic. A full list of the questions asked during the focus groups is provided in Multimedia Appendix 1; however, only a subset of these questions is the subject of this paper. Participants were asked to imagine the concept of a comprehensive Internet resource for mental health and to discuss their attitudes toward this resource, and what content it should contain. This was designed to elicit spontaneous ideas about the potential functions and features of a virtual clinic. Screenshots of other Internet resources were provided to students to provide a context for the discussion. Next, specific prompting questions were asked about the potential functions and features discussed by the participants (eg, “How do you think students would want to connect with other students?”). Focus group questions also addressed other issues including participants’ preferences for help seeking, and their ideas about usage, engagement, and dissemination of the virtual clinic. These data are reported elsewhere.

The primary facilitator (LF) was a female researcher and registered psychologist at the National Institute for Mental Health Research. There were two research assistants (AG and JC) that were present to provide assistance and take field notes during the focus groups. The focus groups were also audio recorded. On arrival, participants were provided with an information sheet to read and a consent form to sign, as well as a short demographic questionnaire (age, gender, number of years of study, study discipline, and domestic versus international student status). At the beginning of each focus group session, the primary facilitator provided a brief introduction to the study and information about the purpose of the focus groups, confidentiality, and voluntary participation. The duration of each session was approximately 1.5 hours.

### Analysis Strategy

Data were analyzed using thematic analysis in NVivo 10 by the first (LF) and third (JC) authors. Participants’ statements in response to each question were coded using a grounded theory approach [22], whereby similar concepts were grouped together into themes. The themes that emerged during discussion of each question are described below under each predetermined topic, and are ordered by relative importance, as determined by the number of comments, as well as the volume and quality of discussion associated with each theme. Direct quotes are used to illustrate the emergent themes and participants are identified by a number and their gender (eg, 1F = Female, participant number 1).

## Results

### Participants

Of the 19 university students who participated in the focus groups, 10 were female. The mean age of the sample was 21.6 years (range 19-24 years) and the mean duration of their tertiary education was 3.1 years (range 1-5 years). Most participants were domestic students (12/19, 63%) from a range of study disciplines (arts, law, commerce, engineering, science, music, and combined degrees from those faculties). Participants were not recruited based on mental health status; nor were they asked to discuss their own mental health during the focus groups. No participant dropped out of the focus groups, although one student left a group early due to personal commitments.

### Topic 1, the Utility and Acceptability of a Virtual Mental Health Clinic for Students

Question, What do you think about the idea of a space online which offers pretty much anything students might need for their mental health?

There were four main themes that emerged during discussion of this question: (1) the virtual clinic as a centralized help source, (2) the desire for professional/human input in the virtual clinic, (3) concerns regarding privacy and uptake, and (4) restriction of the virtual clinic to the university student population.

### Virtual Clinic as a Centralized Source of Help

Participants viewed the virtual clinic as an ideal solution to the problem of widely dispersed, decentralized mental health resources on the Internet,

Everything’s scattered at the moment and if you had something that was bad enough that you knew to go there and then it catered for everything...I think something like that would be so good.

1F

Participants believed that a virtual clinic would make it easier and more convenient for students to find existing resources and help for mental health problems,

Just finding out what services are available for you can be really time-consuming.

2F

[A virtual clinic would be] so convenient as well because you don’t have to go to every place for different little informations (sic). You can just do it in one place.

9F

They also believed that a virtual clinic would be a good first point of call for students to seek help without fear of being stigmatized,

Like for someone who is, I don’t know, needs help but...because of stigma, then, and if you go to website that pretty much has everything covered, it’s, it’s a good place to start for them.

3F

### The Desire for Professional/Human Input

Some participants commented that an Internet clinic may encourage self-diagnosis (which could be inaccurate and potentially dangerous) and believed that it was necessary for professionals to be involved in the clinic to provide personalized feedback and support,

You might think you present with like, one set of symptoms but then like a psychiatrist or like a psychologist might look at you and say like, “well actually you have this type of anxiety or you’ve got these things”. So yeah, you need a professional to help you...

2F

Several participants indicated that they preferred to talk to someone rather than write about their problems on the Internet,

I find with things that sort of have strong emotional content, I much prefer talking than like, writing.

2M

This preference was mainly due to concerns about not being understood or difficulties in communicating via text when other nonverbal information is absent,

I actually get quite anxious when I write about it and I think “oh what if that person doesn’t understand”, whereas talking, you know, there’s sort of a lot more cues and things you can go on.

2M

### Concerns Regarding Privacy and Uptake

Some participants indicated that they would be hesitant to disclose personal information when using a virtual clinic, especially if the service was linked to the university,

If it was something student-based at a university that provided like everything, I’m not sure how much information I’d want to be giving.

4F

Additionally, some participants expressed concerns about the ongoing utility of a university virtual clinic,

Well, you’d go there once and it’s really helpful but you won’t keep going back.

3M

Participants indicated that user-friendly language, regular content updates, interactivity, and easy navigability would be key features to promote the, “stickiness” [4M] or repeated usage by students of a virtual clinic. Several participants indicated that videos could be used to engage students,

I find that students nowadays enjoy watching videos as opposed to reading long paragraphs of text.

1F

### Restriction of the Virtual Clinic to University Students

Some participants were concerned that restricting the virtual clinic to university students may ignore the broader contexts in which students operate (eg, as men, women, young adults),

A lot of people struggle with these issues, you know, at university and elsewhere.

2M

A participant felt that a university-focused virtual clinic might promote the idea that mental illness is exclusively associated with being a student,

You know, I think it’d be more normalizing for mental illness to say “Hey, it’s not just students who experience this, everyone has it”.

1M

### Topic 2, Potential Features of a Virtual Mental Health Clinic

Question, What should be in this space and what should it offer participants?

There were three themes that emerged from discussion of this question: (1) information, (2) access to professionals, and (3) peer-to-peer interaction.

### Information

Provision of information was suggested as an important feature of the virtual clinic. Several subthemes emerged during this discussion: (1) well-being tips, (2) tailored information, (3) information about symptoms, and (4) information about how to help friends.

### Well-Being Tips

Participants felt that the virtual clinic should not only focus on treating mental illness, but also on providing information about how to prevent illness and maintain mental well-being,

Maybe not just an emphasis on treating existing conditions but general well-being.

2F

Participants also wanted information about how to maintain a balanced lifestyle as a student,

Information about balancing lifestyle, about balancing your study with your life.

5M

Motivational messages, especially those that provide positive reinforcement for help seeking, and stories of recovery from people with experience of mental health problems were also viewed as beneficial and reassuring.

### Tailored Information

It was important to participants that the information was tailored to their needs; for example, their area of study, gender, and sexual orientation. A participant felt that the virtual clinic should offer information about issues that are of specific relevance to the university population, such as exam stress, homesickness, and university processes for offering academic adjustments to students experiencing mental health problems,

Information...for issues, that specifically affect uni students would be good.

5F

### Symptom Information

Participants wanted the virtual clinic to contain information about how to recognize if they were experiencing a mental health problem, as well as the causes and symptoms of common mental disorders,

I think basically all the symptoms you would have to look out for...and information on, about, each illness.

6F

Participants also wanted “technical literature” [2M] (ie, scientific studies) on symptoms, consumer experiences, and treatment.

### How to Help Friends

It was important to some participants that they received information regarding how to help friends and family with mental health problems. In their experience, someone experiencing a mental health problem would typically speak to their friends first, and therefore, some participants felt it necessary to know how to respond to these situations,

So that in a way we’re more aware of, sort of like, we make sure that we look after our friends as well.

6M

### Access to Professionals

Access to mental health professionals was considered by many participants to be a necessary component of the virtual clinic. To prompt discussion, participants were specifically asked: “Who would these professionals be?” and “How do you think students would want to access help from a professional?”.

### Types of Professionals

Psychologists, counselors, and youth workers were the most preferred types of mental health professional. Other suggested professionals included social workers and psychiatrists. Participants generally felt that psychiatrists and general practitioners/primary care physicians were not well suited to provide help on the Internet, as they are more focused on prescribing medication and writing referrals, respectively. Participants also felt that it was important to provide options to students in terms of access to different types of professionals,

Good to have a variety of options, for example if you have a bad experience...you want to have that other option as well.

7M

and,

Because you have different degrees of mental illnesses and they can help in different ways.

7M

Additionally, participants felt that it was important for professionals involved with the virtual clinic to receive training to manage issues that are relevant to specific groups (eg, sexual identity in young people).

### Methods of Connection With Professionals

The ability to use the virtual clinic to make appointments to see a mental health professional, either face-to-face or online, was viewed favorably. However, participants varied in their preferences regarding methods of connection with professionals. Online chat, phone, video-calling (eg, through Skype), email, “query boxes”, and forums were suggested by participants as possible options. Some participants viewed these methods as less confronting than connecting with professionals face-to-face,

I think that’s a good idea, instead of going to the counseling center in person, you could do it online so you feel more comfortable sharing details that way.

7F

However, the ability to speak face-to-face with a professional was also viewed positively by participants, even if this contact was initiated by online communication,

It would be nice to kind of have an email or something first to know who I’m communicating with before I meet them face-to-face.

1M

Many participants felt that video-calling could be too intrusive,

It’d be a little bit overwhelming to actually see someone like talking on a screen.

6M

It would be weird because it’s like inside your home.

6F

and,

Yeah, it’s like your personal space.

2F

Although offering access to professionals was seen to enhance the credibility of the virtual clinic, participants had reservations about the effectiveness of providing therapy online compared to in-person,

Would an hour counseling someone online be as effective for them as an hour spent counseling someone in-person?

1M

### Peer-to-Peer Interaction

Participants suggested the capability for peer-to-peer interaction as a useful component of the virtual clinic. There were two major themes that emerged during this discussion: (1) perceived benefits, and (2) privacy issues.

### Perceived Benefits

Participants felt that online peer-to-peer support could be beneficial in a number of ways. For example, it could reduce stigma through discussion of mental illness, foster a sense of community among students, and allow students to share personal stories of recovery, which was viewed as having a positive effect on those who experience mental health problems. The ability to connect with other students who have had similar experiences was viewed favorably,

I think like it’s a really good idea, um, having...like-minded kind of people, getting to see their stories and then it’s “oh ok, it’s not just me”, things like that.

8F

Incorporating a social, peer-led element into the virtual clinic was seen as necessary to promote engagement with the service,

I guess socializing helps keep it active and I guess you want it to be a much more active than passive space.

3M

The ability to organize or advertise group activities, such as meditation or relaxation groups through the virtual clinic was also noted as a helpful way for students to cope with stressful events like exams.

### Privacy and Security Issues

Participants highlighted security of information as a priority in their discussion of peer-to-peer interaction,

I think knowing that your information isn’t going to feedback elsewhere–whether it’s to academics or family–like knowing that your information you shared is secure.

1F

Participants were also concerned that other students could recognize them. The use of anonymous usernames was suggested by participants as a potential approach to enabling privacy. However, a participant felt that students should be given the option to be anonymous,

I feel like there should be an option rather than only being anonymous. I feel like some people are quite comfortable talking about mental health issues and they don’t mind putting their name out there.

1F

There were three additional questions that were asked to prompt further discussion: (1) “How do you think students would want to connect with other students?”, (2) “What do you think about having moderators to manage the peer support component and who should they be?”, and (3) “How do you think discussion of suicide should be handled?”.

### Methods for Peer-to-Peer Interaction

Participants suggested that a range of options for peer-to-peer contact should be provided in the virtual clinic, including chat rooms, forums, a “question and answer” function, the ability to comment on videos, and providing a space for users to share positive messages or posts. Most participants preferred forums due to their ability to benefit those who read as well as those who comment, and their efficiency in generating multiple perspectives on a single issue or problem. With moderation, forums were perceived by participants to be safer than private chats in which monitoring could be impractical or unfeasible. Conversely, as forums are not synchronous, some participants felt that users may lose interest in them or not access the help they need in a timely manner,

People might just lose interest or not think that their issue’s going to be solved as quickly as they need.

8F

### Moderation Issues

The presence of a moderator to monitor discussions between participants was seen as vital to ensure safety within the peer-to-peer component of the virtual clinic. Participants expressed concern about the potential for online bullying, exacerbation of distress, or encouragement of unhealthy behavior in forums. Trigger labels, allowing users to “flag” posts containing distressing content, and the ability to report abuse were suggested as helpful solutions. Participants were also largely in favor of,

clear, well-established

3M

and,

concrete

3M

guidelines for use of the forum,

I think having a moderator who has set up these rules and is willing to implement sanctions if people contravene those rules is very important.

4M

When discussing who they would consider to be appropriate moderators, participants preferred either mental health professionals or those who have had a significant amount of experience using the forum (eg, senior members).

### Discussion of Suicide

While participants recognized that there may be dangers associated with discussion of this topic, they felt that banning discussion of suicide would be isolating, stigmatizing, and may act as a barrier to help seeking,

If it wasn’t allowed then it would be quite a deafening silence.

8M

However, participants noted that discussions about suicide should be,

very heavily moderated

4F

supportive, focused on help seeking, and nonjudgemental,

That people aren’t...being stigmatized or whatever for...contemplating suicide or something like that.

4M

Participants believed that discussion of suicide would need to be closely monitored, and that,

any posts that are potentially dangerous to other users [should] be deleted.

4M

## Discussion

### Principal Findings

Overall, participants viewed the concept of a virtual clinic favorably as an additional option for the provision of mental health services. It was seen as a particularly suitable platform for consolidating online mental health resources and providing help to students who may be reluctant to access other services due to stigma. A previous survey of university students found that the availability of vast amounts of information and the ability to seek help without fear of embarrassment were among their top reasons for using the Internet for mental health support [23]. Participants expressed a clear desire for a virtual clinic to involve human support in some capacity, primarily for credibility and safety reasons. Some students expressed a preference for access to professionals because they were concerned about their ability to communicate their feelings through writing. Similar views were reported in a study asking students to contrast Internet (nonhuman) and face-to-face mental health care [23]. Commonly cited reasons for preferring face-to-face care in this study included the perception that it is more personal, reliable, and conducive to building mutual understanding.

Participants also expressed concerns about the privacy of their personal information and who would have access to it. This may be related to high rates of perceived stigma among university students [6], or fear among students that disclosure of having experienced a mental disorder may be linked to their academic records [24].

When asked about the potential features of a virtual clinic, participants expressed a clear desire for the availability of centralized information, access to professionals, and peer-to-peer support. Participants expressed a strong need for mental health information, which is unsurprising given that reading informational websites is one of the most highly utilized and preferred methods of accessing mental health support on the Internet among university students [25]. Regarding participants’ preferred topics for information, they expressed a preference for information about general well-being, symptoms of mental disorders, how to help friends, and issues related specifically to university students, such as homesickness and study stress. Similarly, work-life balance, time and stress management, coping skills, and anxiety were among the top rated topics of interest reported in a survey that assessed the views of university students toward an online mental health intervention [26].

In terms of the types of professionals that participants wanted to access, participants preferred psychologists and counselors, particularly those with skills and experience relevant to the university student population. Participants also wanted the ability to make appointments with professionals through the virtual clinic, and suggested several methods of connecting with professionals, including by chat, email, forums, and telephone. Use of video-calling or Skype was not rated favorably by some participants because it was considered to be too intrusive. It appeared from the discussion that although participants expressed a strong preference for connecting with professionals, they wanted to do so on their own terms, using methods that they perceived as less confronting.

Participants discussed peer-to-peer interaction positively, as a method for enabling social support and sharing of personal stories of recovery. Similar views have been expressed by young people in a case study of the development of an online community forum for young people [27,28]. A previous study of an online forum for university students found that users benefitted from being able to identify with the experiences of others, which helped them to cope with feelings of loneliness [29]. The majority of participants indicated a desire to remain anonymous, which has been previously found to be extremely important to users of online mental health forums [30]. A frequently raised issue during the discussion of forums was the importance of ensuring the safety and privacy of participants. Participants expressed concern about the possibility of bullying or exacerbation of distress in forums, and that this could be circumvented by providing a clear set of rules governing use of the forum and the presence of an experienced moderator. Higher levels of moderation may be required to ensure the safety of users, in light of evidence suggesting that lower levels of moderation were associated with higher levels of depressive symptoms and symptom contagion effects among users of an online self-harm support group [31]. Participants believed that banning discussion of suicide could be stigmatizing. However, they acknowledged that discussion of suicide would require close monitoring and should focus on help seeking. In practice, balancing concerns about distress and safety issues with concerns about the perpetuation of stigma is likely to prove highly challenging.

### Limitations

There are several limitations associated with the current research that require consideration. Participants were not selected on the basis of current or previous experience of a mental disorder, and their mental health status was not assessed. It is unclear to what extent the views of students without mental health problems are applicable to those of students experiencing a mental illness. However, several participants disclosed having previously experienced mental health problems during the focus groups and framed their responses to the questions in terms of their own experiences. Moreover, although the virtual clinic is designed to target mental health, its purpose is to be disseminated universally to the entire student population, to assist not only those with current mental health problems, but to provide prevention tools to students without symptoms. Given that participants self-selected to participate and this study was conducted in one university setting, the views expressed may not represent the views of ANU students generally, or students from other universities. However, it is arguable that reasonable diversity in terms of gender, domestic and international student status, and discipline of study was obtained in the sample to represent the wider student body. Finally, we did not ensure that participants who invited other students to attend were allocated to different groups, as composition of the groups was based purely on when participants were available to attend. Preexisting relationships between group members may have potentially biased the discussion.

In addition, these focus groups were conducted in the first, exploratory stage of the design and development of a virtual clinic, and some participants found it hard to conceptualize what a virtual clinic would actually involve. Later stages of the project will include further student involvement through testing and discussion of functional and nonfunctional prototypes. It may be that these feedback mechanisms, which offer greater context, will generate additional, differing, or expanded student perspectives.

### Implications

The results of this study suggest that although university students are generally in favor of a virtual clinic, they have reservations about privacy, trustworthiness, and the ability of online interventions to deliver the same quality of care as other services. Thus, online services targeting university students should be built on evidence-based principles, and streamline or simplify the help seeking process for students. Moreover, increased awareness of the effectiveness of online mental health interventions is needed among students to increase their confidence in this form of treatment. Privacy concerns and stigma are also major issues for university students, who fear that disclosure of, or seeking help for, a mental health problem may negatively affect their success at university or their subsequent career opportunities. Providing the capability for students to access help privately online may go some way to address this issue. It is also important to ensure that information about privacy and models of information sharing are made explicit to service users and are within their control.

Although participants in this study feared that tailoring a virtual clinic to university students might be stigmatizing, they expressed a clear desire for the functions and content of the virtual clinic to address the issues commonly experienced by university students. Arguably, tailored, relevant content will increase the appeal and likelihood of uptake of an online service for students, and indeed this has been identified as an important priority among end-users of virtual clinics for other chronic health conditions such as diabetes [32]. Moreover, uptake of a service like this within a university environment will require cooperation and buy-in from all levels within the university, including the executive, health and student services, student organizations, heads of faculties and departments, residential halls, and teaching staff. A possible strategy to encourage awareness and uptake among students would be to universally disseminate the virtual clinic as a prevention and treatment tool to students during their orientation to the university, at the start of each semester, and during stressful times throughout the academic year such as exam periods.

Participants also expressed a desire to connect with mental health professionals through the virtual clinic. This raises important issues regarding resourcing and the implementation of university-based virtual clinics over time and in different settings. A balance is needed between the competing demands of making online services scalable, widely implementable, and cost-effective, while meeting the needs and expectations of users for professional human involvement. Models for human involvement need to be flexible and responsive to the availability of professionals in different university environments. Groups within the university such as trained lay-students, consumers, and clinical psychology trainees could provide support, moderation, or clinical services to users, which may reduce the level of mental health professional involvement required.

This paper presents the first step in a larger user-centered design process being undertaken to develop the virtual clinic. The data obtained in this study will be used to develop and build prototypes for testing with students and other stakeholder groups. Feedback from testing sessions will be used to refine the prototypes for further testing and finalize the functionality that will comprise the virtual clinic. Thus, the findings of this research have direct implications for the development of online interventions to improve the mental health of university students. Despite the impact that untreated mental health problems can have in emerging adulthood, mental health problems still remain undertreated in this vulnerable group. A university-specific virtual clinic may address some of the help seeking barriers that students experience, and allow universities to improve rates of help seeking among their students.

## Appendix

Multimedia Appendix 1 Focus group questions.

## Abbreviations

ANU: Australian National University
CRC: Cooperative Research Centre
