# Supplementary material for: A Virtual Mental Health Clinic for University Students: A Qualitative Study of End-User Service Needs and Priorities
Source: JMIR Ment Health. 2015 Feb 11;2(1):e2. doi: 10.2196/mental.3890 (PMC4607392; doi:10.2196/mental.3890)
Supplement: Multimedia Appendix 1 [file mental_v2i1e2_app1.pdf]

Appendix 1

Focus group questions
1. As a university student, what would you do if you thought you had an emotional or mental health problem?<br>2. What would you advise a friend to do if they thought they had an emotional or mental health problem?<br>3. In an ideal world, what kind of mental health help do you think students would want to receive?<br>4. What do you think about using the Internet for getting support for emotional or mental health problems?
5. What do you think about the idea of a space online which offers pretty much anything students might need for their mental health?
6. What should be in this space? What should it offer students?
7. Would students want to be able to access information about emotional and mental health problems using this space?<br>8. What kind of information would students want to receive?
10. Through the space, do you think students would want the ability to connect with other students who may be experiencing stress, emotional or mental health problems?<br>11. How do you think students would want to connect with other students?<br>12. What do you think should be done to make sure that this peer support component is safe for users?<br>13. What do you think about having rules that specify what is appropriate to talk about?<br>14. There is potential for sensitive topics like suicide to be discussed in these types of peer support networks. How do you think discussion of suicide should be handled?<br>15. What do you think about having moderators to manage the peer support component? Who should they be?
22. Do you think students would want to have access to mental health professionals (such as counsellors or psychologists) through this space?<br>23. Who would these professionals be?<br>24. How do you think students would want to access help from a professional?<br>25. If face-to-face, do you think students would want to be able to make an appointment online?

26. Would students want the professional to be able to see the information they have entered into the online space?<br>27. Do you think it might be helpful for students to have access to a forum to interact with professionals?
There are a number of self-help programs that people can access online for mental health problems. For example, MoodGYM is a program that provides self-help therapy for depression. It teaches you the same kinds of skills that talking to a psychologist would teach you. The program is automated – it doesn't involve a person at the other end.<br><br>34. Do you think students like yourselves would use a program like this?<br>35. What do you think about self-help programs like this being available as part of the space?<br>36. Sometimes these programs are used with a support person. Do you think students like yourselves would want someone to support them to use an online self-help program?<br>37. Who would this be?<br>38. How often/under what circumstances do you think students might want support?<br>39. How might students want to be contacted?
40. Should the space be used for preventing mental health problems?
41. What about its use by students who are recovering or have recovered from a mental illness?
42. What problems/issues should the space target?
43. If students were to come to the space for help, what information (about students) should the system collect?<br>44. How should the space collect this information?
45. Do you think students would want feedback from the space after this information is collected?<br>46. In addition to giving feedback, what else should the space be able to do with students' information?
47. Is there anything you think students wouldn't want the space to ask them about?
48. In order to do some of what we've been talking about and to tailor the space to students' needs across time it would be necessary for students to register to use the space via a username and password. During the

	registration process, what personal information would students like yourselves be comfortable providing?
49.	Should parts of the space be available without having to register?
50.	To what extent do you think students would like the space to interact with them once they have registered?
51.	How would students want to navigate the space?
52.	When would students be likely to use the space?
53.	How can students give feedback about the parts of the space that were helpful for them and parts that weren’t helpful?
54.	How do we get the space out to students?
55.	Are there any reasons why students might not want to use the space?
56.	What should we call the space?
57.	How do you think students would prefer to access the space?
